# The Highs and Lows of Making a Bucket List—Quantifying Potential Mosquito Breeding Habitats in Metropolitan Backyards

**DOI:** 10.3389/fpubh.2017.00292

**Published:** 2017-11-06

**Authors:** Ram Sharan Lamichhane, Peter J. Neville, Jacques Oosthuizen, Kim Clark, Samir Mainali, Maria Fatouros, Shelley Beatty

**Affiliations:** ^1^School of Medical & Health Sciences, Edith Cowan University, Joondalup, WA, Australia; ^2^Medical Entomology, Environmental Health Directorate, Public Health Division, Department of Health, Perth, WA, Australia; ^3^Environmental Health Officer, Town of Bassendean, Perth, WA, Australia

**Keywords:** backyard, container-breeding mosquitoes, *Aedes notoscriptus*, urban development, surveillance carbon dioxide traps, metropolitan, australia

## Abstract

While the development of land for residential housing along the Swan and Canning Rivers in Perth, WA, Australia has reduced natural mosquito breeding sites, the role of backyard container breeding remains a relatively unknown factor. Local Governments responsible for these areas focus management and control efforts on low lying, tidally driven mosquito habitats to control *Aedes vigilax* (Skuse) and *Aedes camptorhynchus* (Thomson) mosquitoes in an effort to reduce both the nuisance and disease risk to residents. In spite of their efforts, Local Governments continue to receive complaints regarding mosquito nuisance, even when environmental conditions do not favor hatching and development of the two species in the Swan River tidal flats. In this study, 150 backyard inspections were conducted in the residential suburb of Bassendean, Perth, WA, Australia, situated in close proximity to the Swan River tidal plain. The occurrence and species composition of the mosquito fauna found in residential backyards was documented. Of the backyards inspected, 94% were found to possess containers capable of breeding mosquitoes, although only 3% contained mosquito larvae. Nine species of mosquito were collected from containers ranging in capacity from 0.05 to 50 L across the study area. Additionally, encephalitis virus surveillance trapping was conducted within residential properties and compared to the tidally driven natural habitat at Ashfield Flats and a tidally influenced brackish creekline at Bindaring Park. The species composition of the fauna at the three habitat types differed significantly, with *Aedes notoscriptus* (Skuse) dominating residential lots and *A. vigilax* more prevalent at the saltmarsh site. Bindaring Park had an adult composition at the mid-point of these two habitats, reflecting its proximity to both the Swan River and residential lots.

## Introduction

Since European settlement in 1829, approximately 70% of Perth’s Swan Coastal Plain wetlands in Western Australia have been lost through drainage and infill (1, 2). A need for housing in close proximity to the City’s Central Business District led to the development of suburbs along the Swan and Canning Rivers, further degrading and reducing biodiversity of these wetland systems (3). In recent decades, urbanization has continued to claim natural wetlands in metropolitan Perth, with a loss of 1,500 ha between 1996 and 2005 (4).

Part of the underpinning problem has been the continuing expectation of Perth residents to be able to live in single dwellings on land ranging from 0.2 to many hectares in close proximity to the river foreshore (1), posing an ongoing threat to remaining wetlands. Associated with this urbanization, there has been an increase in water infrastructure, with the development of drainage lines, sumps, hidden absorption basins, and constructed swales for water runoff and disposal (5). A study conducted in Kalamunda, located in the Perth Hills, demonstrated that road drainage gullies contributed significantly to mosquito abundance (6). During the dry summer season the gullies receive lawn irrigation runoff and this stagnant water produced in excess of 1,600 mosquitoes per day. The most abundant species were *Culex quinquefasciatus* Say and *Aedes notoscriptus* (7).

In addition, artificial wetlands, often associated with water runoff infrastructure within new suburbs of Perth, are becoming a feature requiring additional management and control of mosquitoes that breed within them (8). Recent advances in Water Sensitive Urban Design have led to the development of a Better Urban Water Management Framework to integrate water and land-use planning (9).

While the loss of natural wetlands has been seen as disadvantageous to biodiversity overall, it has led to some improvements in public health by reducing mosquito and midge breeding habitats. Coupled with this, the loss of habitat for macropods and possums, the host reservoirs for mosquito-borne diseases that affect the area, including Ross River (RRV) and Barmah Forest viruses (BFV) has led to a reduction in the incidence of these diseases.

Although there has been a reduction in wetlands, tidal inundation of remnant saltmarsh habitat fringing the Swan and Canning Rivers, particularly under favorable climatic conditions associated with La Niña weather patterns, still support regular and often intense mosquito development that directly affects residents in close proximity to these habitats. Two nuisance mosquito species, namely, the Southern Saltmarsh Mosquito (*Aedes camptorhynchus*) (Thomson) and the Summer Saltmarsh mosquito (*Aedes vigilax*) (Skuse) (10), are frequently associated with these habitats. Both species are known vectors of RRV and BFV (11–13).

Local Government Environmental Health Officers (EHOs) are responsible for monitoring and treating mosquito-breeding sites, primarily of Government-owned land, and public open space. While these sites are well known, with regular monitoring and treatment during the peak mosquito breeding seasons, EHOs are limited by a lack of legislation in their capacity to access and, where appropriate, apply treatments on privately owned land. As urbanization increases, a new risk associated with residential lots is emerging. Container mosquito species may be contributing to the overall nuisance and possible disease risk to residents. A number of mosquito species that once bred in tree holes or rock crevices are adapting to residential housing by occupying container habitats and manmade structures. Further, water harvesting by residents in response to warmer and drier weather patterns (14–16) is contributing to the mosquito problem with significant breeding occurring in rainwater tanks.

This study was undertaken in the Town of Bassendean, approximately 12 km east of the Perth Central Business District in Western Australia. The study location was chosen due to its proximity to two documented mosquito-development habitats, namely Ashfield Flats and Bindaring Park. Ashfield Flats is a 40-ha tidal-driven saltmarsh mosquito-breeding habitat located on the northern bank of the Swan River that produces large numbers of the Southern Saltmarsh mosquito (*A. camptorhynchus*) in spring and autumn months, while the Summer Saltmarsh mosquito (*A. vigilax*) breeds in large numbers during the summer period. Bindaring Park, on the other hand, is a brackish creek, that is driven through water runoff from surrounding residential properties and street drains breeding *Culex annulirostris* and *C. quinquefasciatus* mosquitoes. To date, these sites have been held responsible for the nuisance risk posed by mosquitoes and the high number of public complaints received by the Town of Bassendean each year. This study aimed to survey container-breeding habitats associated with residential lots in an effort to quantify the role of backyard container mosquitoes and their contribution to nuisance and disease risks to residents. It was, therefore, hypothesized that backyard breeding contributes significantly to the mosquito density of the residential areas of Bassendean located close to the Swan River.

## Materials and Methods

**Figure 1:**
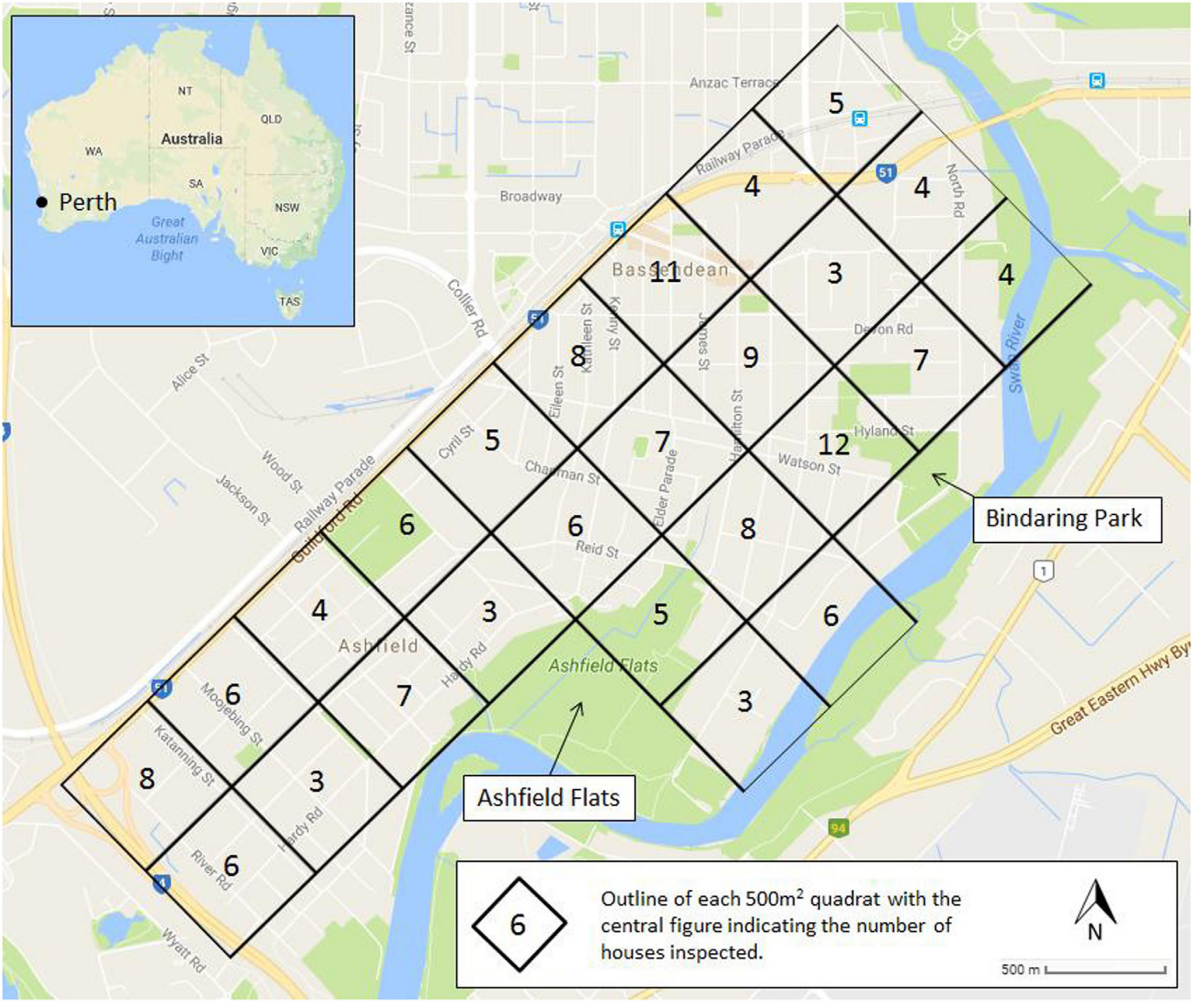
The study area within the Town of Bassendean, Perth, WA, Australia showing the 25 quadrats and the number of household inspected within each quadrat (central figure within each quadrat).

To evaluate the role of backyard breeding in the Town of Bassendean, a survey of 150 residential house lots was undertaken from early December 2015 until the end of March 2016, which is the time of year when most mosquito complaints are received. The study area was divided into 25, 500 m^2^ quadrats bordered by the Tonkin Highway to the west, Guildford Road to the north, and the Swan River to the south and east (Figure 1). Microsoft Excel was used to randomly designate the order in which the 25 quadrats were to be sampled, and the number of households to be inspected (between 3 and 12), until a total of 150 residential house lots had been chosen. Prior to the collection of data, the study was approved by the Edith Cowan University Human Research Ethics Committee (Ethics approval number 13843). Researchers wearing “fight the bite” uniforms of the Western Australian Department of Health (DoH) and carrying identification cards approached the selected residential properties and sought approval from the home owners to conduct the survey. Once permission was granted, a systematic inspection of the yard was conducted to identify and record all mosquito development habitats, both natural and manmade. Data on development habitat/container, presence of stagnant water, and/or the presence of larvae/pupae was recorded. If present, larvae were collected with larval dippers or turkey basters and placed in labeled vials for transport to the DoH Medical Entomology laboratory. Collected larvae and pupae were reared to adults and identified using taxonomic keys (17) and stereo-microscopes.

Additionally, encephalitis virus surveillance carbon dioxide (EVS CO_2_) traps were deployed at one of the inspected residences within each quadrat throughout the study area to collect adult mosquitoes from surrounding properties. The EVS CO_2_ traps were baited with dry ice and set each afternoon following backyard inspections. The traps were collected the following morning. Adult mosquitoes were killed on dry ice to preserve the sample and species were identified using taxonomic keys (17). EVS CO_2_ traps were deployed every 3 weeks during the study period, at each of the natural mosquito development sites (Ashfield Flats and Bindaring Park) to compare the abundance and species composition of the mosquito fauna at known breeding locations within the study area. The House Index, Container Index, and Breteau Index were calculated in accordance with World Health Organization Guidelines (18).

Determination of species profile differential between backyards and the two known mosquito development habitats as indicative of the occurrence of backyard breeding was planned using a chi-square test of the relative species distributions. Further diversity statistics, including species richness, Shannons Diversity, and Evenness were calculated to compare species composition at the three habitat types.

## Results

Of the 150 households inspected, 141 (94%) were found to have at least one container habitat capable of supporting mosquito breeding. However, this value fell to a House Index of 26% for households with larvae/pupae present within water holding containers. A total of 1,711 potential mosquito breeding habitats were identified with 62% (1,060) dry at the time of inspection, a further 34.2% (586) were holding water without mosquito larvae or pupae present, and a Container Index (number of containers holding larvae/pupae) of 3.8% (a total of 65 containers). An average of 11.4 potential mosquito breeding containers were found in each backyard inspected across the study area while a Breteau Index of 43.33 was obtained for the number of positive containers per 100 houses. Forty-four (29.33%) of the homes surveyed were positive for active mosquito container development sites.

**Figure 2:**
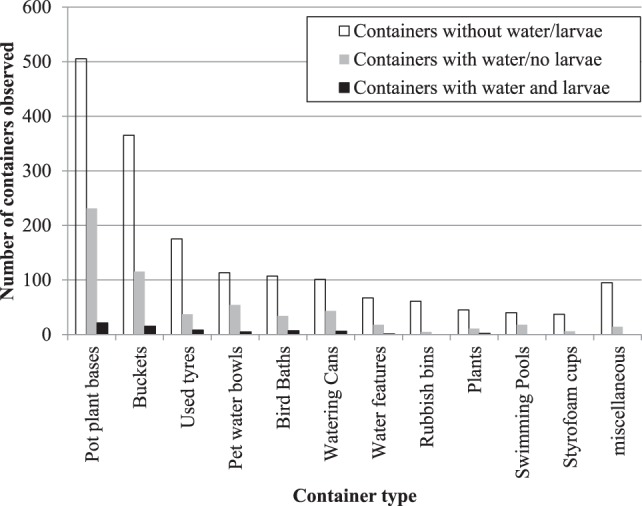
Type of containers with the potential to breed mosquitoes identified from backyard inspections within the town of Bassendean, Perth.

Overall, the most common container types with the potential to breed mosquitoes included pot plant bases (505 containers); buckets (365); used tires (175); pet water bowls (113); bird baths (107); watering cans (101); water features (67); domestic rubbish bins (61); water holding plants including tree holes (45); swimming pools (40); styrofoam cups (37); and other miscellaneous container types (95) (Figure 2).

A similar trend was observed in the abundance of containers that were actually breeding mosquitoes (presence of mosquito larvae and or pupae) at the time of inspection (Figure 2). The most common container types included pot plant bases (21 containers); buckets (15); used tires (8); bird baths (7); watering cans (6); pet water bowls (5); water holding plants (2); and a single water feature (1). While the types of containers with mosquitoes’ present was similar in proportion across container types, larvae, and/or pupae were not found within rubbish bins or styrofoam cups and the swimming pools inspected were well maintained with no evidence of mosquito breeding.

A total of 378 adult mosquitoes composed of nine different species were reared from larvae collected from backyard containers. There was higher variability in the number of larvae collected from the smaller containers (plants, pot plant bases, pet water bowls, and used tires) as compared to the larger containers, with smaller containers holding more larvae than the larger container types.

**Figure 3:**
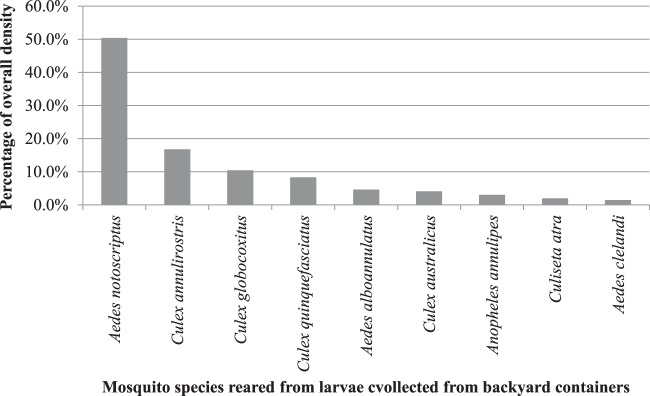
Overall percentage density of mosquitoes from larvae collected from backyard containers across the study area.

The most commonly reared mosquito was the common container-breeding mosquito (*Aedes notoscriptus*) accounting for approximately 50.3% of the larvae collected (Figure 3). This was followed by *C. annulirostris* Skuse (16.7%); *Culex globocoxitus* Dobrotworsky (10.3%); *C. quinquefasciatus* (8.2%); *Aedes alboannulatus* (Macquart) (4.5%); *Culex australicus* Dobrotworsky and Drummond (4.0%); *Anopheles annulipes* Walker (2.9%); *Culiseta atra* (Lee) (1.9%), and *Aedes clelandi* (Taylor) (1.3%).

**Figure 4:**
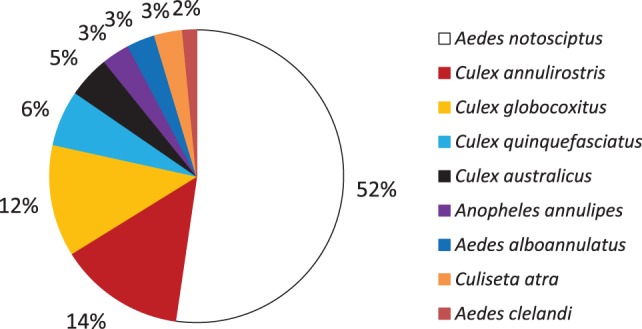
Percentage of containers found breeding each species of mosquito collected across the study area.

**Figure 5:**
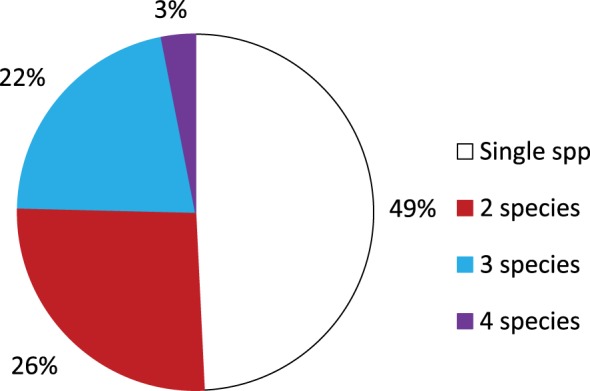
Percentage of containers breeding a single, two, three or four species of mosquito within individual containers.

*Aedes notoscriptus* were found in 52.3% of the containers holding larvae (Figure 4). This was followed by *C. annulirostris* (14%); *C. globocoxitus* (12%); *C. quinquefasciatus* (6%); and *C. australicus* (5%). *A. annulipes, A. alboannulatus*, and *C. atra* were found breeding in 3% of containers, while *A. clelandi* was found breeding in only 2% of containers. Approximately, 50% of containers were found with only a single species present (Figure 5); while 26 and 22% contained two and three species, respectively. Only 3% (2) of the containers were found to contain four species, and in both instances the containers were pot plant bases.

**Figure 6:**
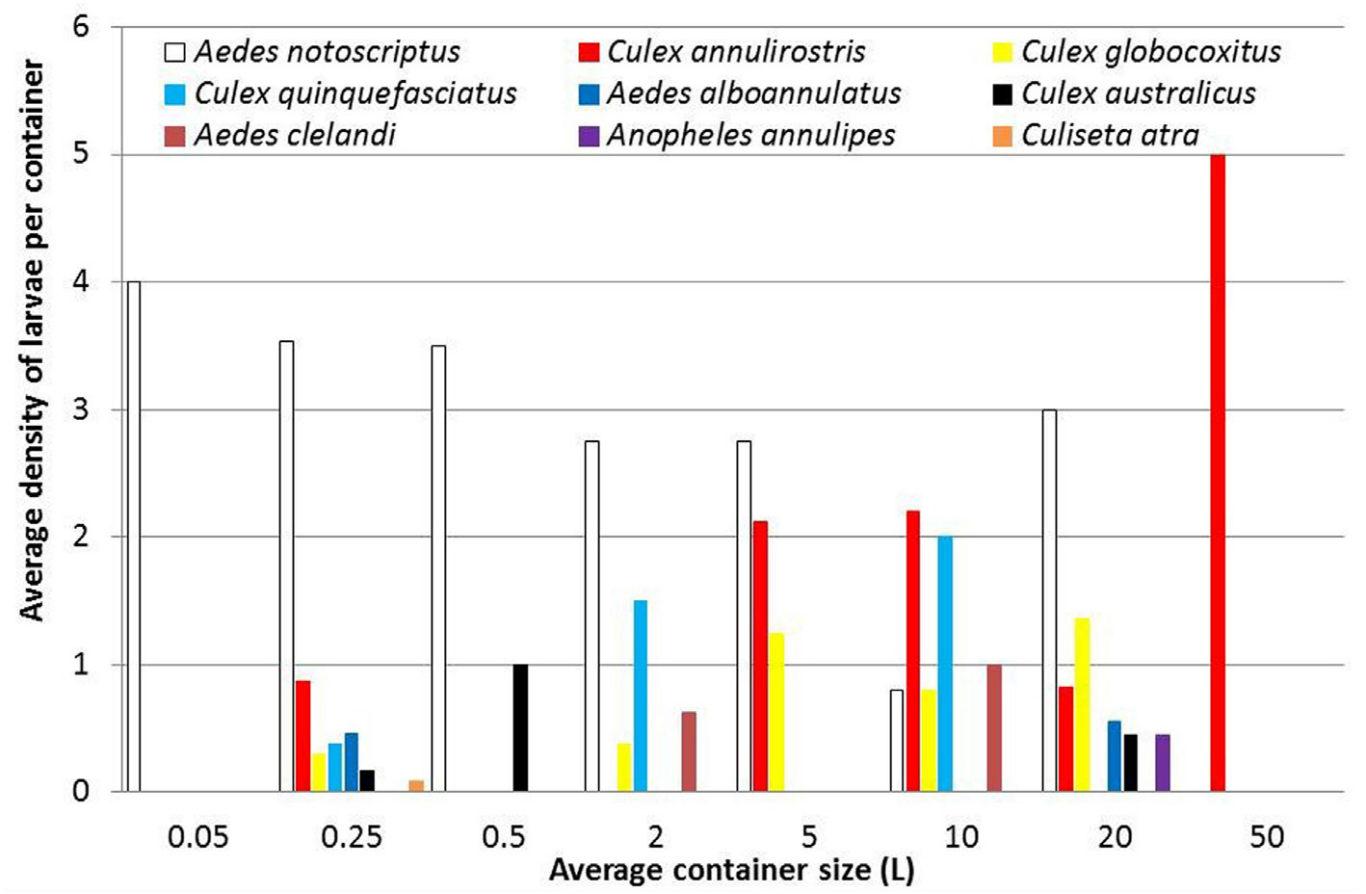
The average density of mosquito larvae collected from backyard containers, including 0.05 L (water holding plants); 0.25 L (pot plant bases); 0.5 L (pet water bowls); 2 L (used tires); 5 L (Bird baths); 10 L (watering cans); 20 L (buckets); and 50 L (water features).

*Aedes notoscriptus* were found to breed in all container types excluding the largest container habitat (water features; 50 L) in roughly equal densities across the study area (Figure 6). This contrasts with *C. annulirostris* that was found predominantly in container sizes holding five or more liters of water. *C. globocoxitus* were found in most container size classes, excluding the smallest and largest containers. Similarly, *C. quinquefasciatus* were generally found in the middle container size categories, although no clear pattern could be detected. Furthermore, no clear pattern could be discerned in container size preference for *A. alboannulatus* [only being found in pot plant bases (0.25 L) and plastic buckets (20 L)]; *A. clelandi* [only occurring in used tires (2 L) and watering cans (10 L)]; nor *A. annulipes* or *C. atra* that were only detected in one container type [plastic buckets (20 L) and pot plant bases (0.25 L), respectively].

**Figure 7:**
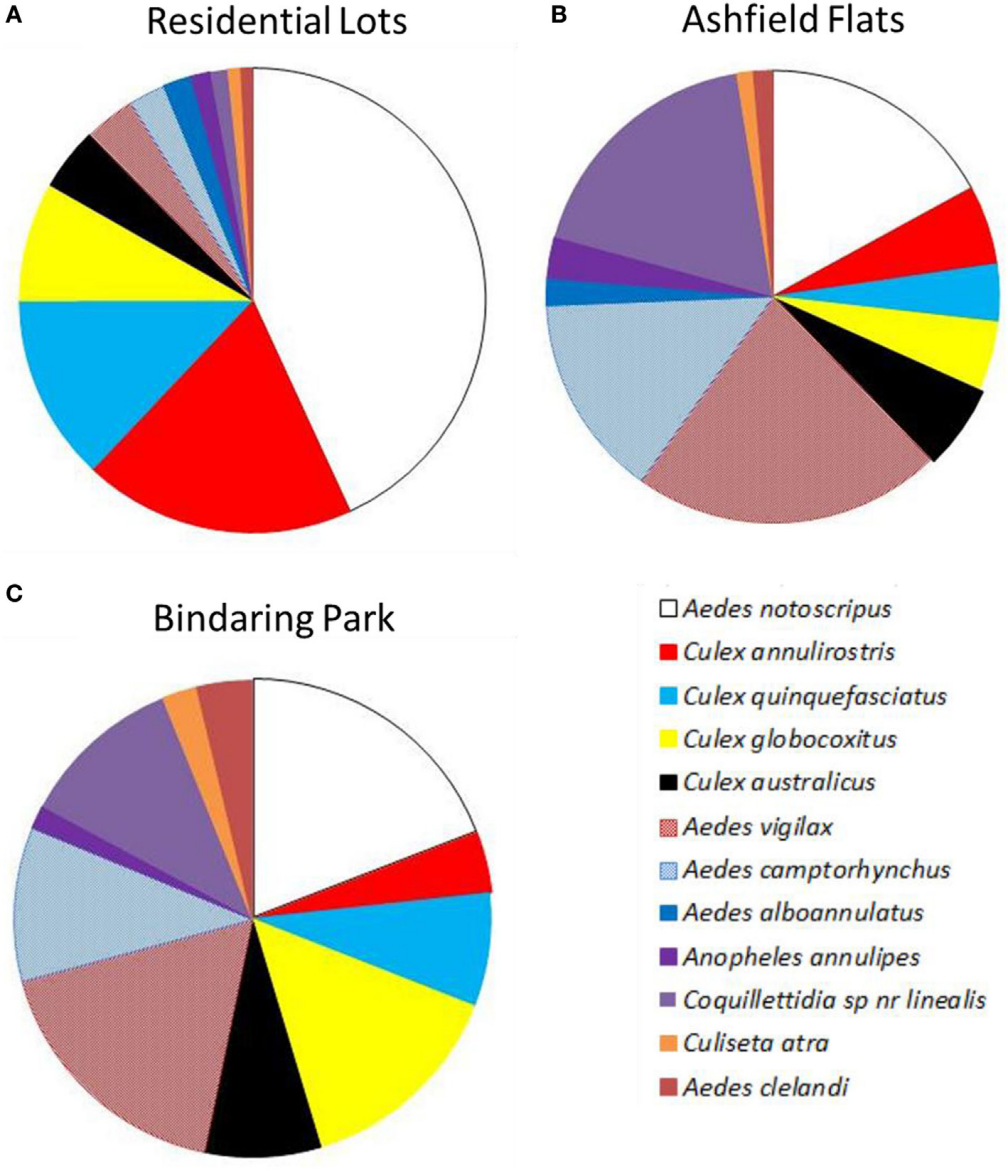
Average composition of adult mosquito species per trap night collected in encephalitis virus surveillance carbon dioxide traps across the study area, including **(A)** residential sites, **(B)** Ashfield Flats, and **(C)** Bindaring Park.

Results from EVS CO_2_ traps collected a similar number of species regardless of location between residential properties (12 species), Ashfield Flats (12 species) or Bindaring Park (11 species) (Figure 7). However, the average density of individual species collected per trap night differed markedly across the three habitats types. Adult *A. notoscriptus* dominated backyards with an average of 36.7 adults per trap (Figure 7A). This was followed by *C. annulirostris* (16.1 adults per trap); *C. quinquefasciatus* (11), and *C. globocoxitus* (7). The saltmarsh mosquitoes were collected in low abundances from residential properties with *A. vigilax* and *A. camptorhynchus* reaching average densities of 3.0 and 2.2 adults per trap, respectively.

In Comparison, average adult densities at Ashfield Flats were dominated by *A. vigilax* (18.8 mosquitoes per trap night); followed by *Coquillettidia species near linealis* (15.5); *A. notoscriptus* (14.5); and *A. camptorhynchus* (12.3) (Figure 7B). Bindaring Park represented a fauna profile between these two distinct habitat types with a more even distribution of species (Figure 7C). This brackish creek site was dominated by *A. notoscriptus* (16.2 mosquitoes); *C. vigilax* (14.8); and *C. globocoxitus* (12.2), followed by a range of species with medium densities, including *Coquillettidia species near linealis* (9.3); *A. camptorhynchus* (8.8); *C. australicus* (6.8); and *C. quinquefasciatus* (6.5).

A chi-square test comparing the average per trap profile for the seven most common species found across all traps across backyards versus the two known mosquito development habitats was significant at the 0.01 level (χ^2^ df = 12, *N* = 37, *p* < 0.001). In contrast, a chi square test comparing the average per trap profile for the seven most common species across the two known mosquito development habitats was not significant (χ^2^ df = 6, *N* = 12, *p* = 0.26).

Diversity statistics compared the three habitat types for adult mosquito assemblages. Species richness was similar across all three habitats with 12, 12, and 11 species recorded for backyards, Ashfield Flats, and Bindaring Park, respectively. Shannon’s diversity statistics where highest at Bindaring Park with a value of 0.84, followed by Ashfield Flats (0.79), while the lowest values was found within residential backyards (0.65). Calculation of species evenness showed a similar trend with the highest value recorded at Bindaring park (0.82) followed by Ashfield Flats (0.74) and lastly residential backyards (0.57).

## Discussion

A survey of residential backyards in the Town of Bassendean, Perth, WA, Australia during the summer of 2015/2016 showed a low occurrence of positive backyard mosquito breeding with a Container Index of 3.8% and a Breteau Index of 43.33%. However, the House Index of 29.33% indicated that larvae/pupae were present in approximately one-third of backyards surveyed. Statistical comparison of backyards and known mosquito development habitats also indicated a significant difference in the backyard species profile as compared to the natural breeding habitats.

Mosquito breeding prevalence in this study was relatively low when compared to findings from other parts of Australia. A study conducted in residential suburbs of Brisbane, QLD, Australia, reported that 26% of containers in backyards were breeding mosquitoes (16). In a survey conducted in the mid 1980s in New South Wales, 83% of domestic or peri-domestic properties were found to be breeding mosquitoes (19). These findings may no longer be relevant as there have been significant changes since the mid 1980s in domestic water storage systems and public information programs have raised awareness among residents in regards to backyard breeding. In Auckland, New Zealand, 34% of surveyed backyards were found to be positive for backyard breeding (20), while in tropical Kuala Lumpur, Malaysia, it was found to be only 24.8% (21). Backyard breeding was found to occur in 38% of properties in Tamil Nadu, India (22), while a study across four villages near Zaria in Northern Nigeria found positive backyard breeding of mosquitoes in containers at a frequency of 16.53% (23). Positive container breeding from backyard surveys in urban developed areas of Rio de Janeiro occurred on 9% of properties (24).

This variance in House, Container, and Breteau Indices may be related to a higher level of awareness driven by public education campaigns in areas with regular incidents of mosquito-borne diseases, including Dengue Fever and Malaria, particularly in countries where these diseases are present. Further, seasonality has been found to highly influence mosquito indices (including the House Index, Container Index and Breteau Index) (25). The low indices indicated by the current study may be influenced by the dry summer experienced in 2015/16 with a total of 63 mm of rainfall during the 4 months of the study (26).

Levels of backyard breeding vary substantially between studies reflecting differences in socio-economic factors, the prevalence of water storage for domestic use, microhabitat conditions (for example, level of shading), and the presence of mosquitoes to colonize container habitats (27, 28). Regional differences in public education and perceived levels of risk associated with mosquito-borne diseases may also drive the behavior of owners of residential properties regarding the availability of containers for mosquito development (29).

In spite of the fact that 94% of backyards in the current study area had the potential for mosquito development within container habitats, 62% of these were dry at time of inspection. Perth received most of its rainfall during the winter months, and during the study period (December through March), Perth received a total of 63 mm of rainfall (26). This may be a possible reason for the reduction in indices compared to other studies throughout Australia. However, the containers found holding water may have been filled or topped up with water from reticulated garden irrigation systems, with most homes in the area having such systems that operate twice a week on an automatic timing system. Further, the role of reticulation in providing a water source was not investigated in the current study and may have influenced eggs hatching and developing in these containers as the water level rose and fell. These reticulated watering systems are fed from the underground water table and it would be interesting to determine if water quality attributes may have influenced rates of oviposition within these container habitats around the home. Further study on the water quality attributes associated with “bore” water compared to rain fed or drinking water and the rates of oviposition success should be considered in future research.

The most common container types breeding mosquitoes were found to be pot plant bases (21 containers); buckets (15); used tires (8); bird baths (7); watering cans (6); pet water bowls (5); plants or plant parts (2); and a single water feature (1). These findings were similar to those of other Australian studies (16 and 19), with the noticeable difference that in Queensland there were many more rainwater tanks, this is likely due to the fact that property owners in Queensland are offered rebates for the installation of rainwater tanks (16) whereas most suburbs in Perth are connected to underground water reticulation systems.

Results from backyard inspections only provide a snapshot of the fauna present at the time of inspection. Numerous physical, environmental, and chemical factors may influence the oviposition of eggs within receptacles. Physical attributes including container size, presence of a cover or lid, and how the container was flooded were found to be key determinants of oviposition (30). Environmental variables, including temperature, light, water depth, turbidity, and the presence of competitors, have been found to influence the oviposition of adult female mosquitoes (31, 32). Similarly, the presence of predators (for example backswimmers) has been demonstrated to reduce oviposition (33–35). Studies on *C. annulirostris* and *Culex molestus* have shown how chemical and microbial mediated products influence egg deposition (36). Additionally, Wong et al. (30) demonstrated oviposition by *Aedes aegypti* (another container-breeding species) to be influenced by conspecific larvae and pupae. Both *C. quinquefasciatus* and *Aedes australis* prefer to oviposit in receptacles that contained, or had contained, conspecific larvae (37), this finding is supported by the fact that most containers in this study bred a single species of mosquito. This may link with competitive pressures which require further research to determine the role of competition in habitat selection by gravid female mosquitoes.

*Aedes notoscriptus* was the dominant mosquito species breeding in backyard containers making up just over 50% of the total number of mosquitoes reared from larvae and it was also the most frequent colonizer of domestic backyard containers (49% of total colonized containers). This species traditionally breeds in tree holes and rock pools, but has adapted to breed in containers within peri-urban and urban environments (17). These findings are supported by other Australian studies, including Trewin et al. (16) who found *A. notoscriptus* contributing 67.4% of the fauna in summer and 58.5% in winter in Brisbane suburbs, while *A. notoscriptus* were found in 61.5% of the containers in NSW, Australia (19). These results clearly demonstrate the adaptation of this species to domestic situations, particularly when the densities of containers in backyards are high. *A. notoscriptus* is known to be a vicious biter of humans, feeding throughout the day with a preference for dawn and dusk (38). Its short dispersal ability, being around 130 m (39), demonstrates the importance of maintaining a backyard free of containers to reduce future populations of this species. *A. notoscriptus* not only contributes a nuisance risk, but it is also a vector of both RRV and BFV, posing a public health risk (40). This species was recorded from all container size categories excluding water features holding on average 50 L of water, perhaps indicating such artificial habitats are not suitable for this species, water features do not resemble natural breeding habitat (tree holes and rock pools) normally colonized by this species. Similar results were found in a study in Auckland, New Zealand with *A. notoscriptus* showing a significant preference for medium sized containers (41, 42). Further, the species has been recorded in high numbers associated with street drainage in Kalamunda, Perth, indicating that these habitats may be providing additional development and harborage sites within the built environment (6) allowing regular colonization of container habitats within residential backyards.

*Culex annulirostris* was the second most abundant species (16.7% of containers) and was found breeding in the larger sized containers identified in the study. This species generally breeds in large temporary waterbodies, flooded grasslands, reed swamps, and it has been recorded in large densities in sewage effluent, organic waste water, and stormwater drains (43). These preferred habitats may explain why this species was found in the larger containers in this survey. *C. annulirostris* is also a known vector of RRV and BFV (40, 44–46) and poses a public health risk to residents.

Interestingly, the third most common species collected from backyard containers was *C. globocoxitus* at 10.3%. This species is associated with open swamps and is often found breeding in brackish water (17). It prefers to feed on birds, thus does not pose a nuisance or disease risk to humans (47). The tidally influenced Bindaring Park creekline provides an ideal breeding site for this species, as demonstrated by its dominance from adult CO_2_ trap catches at this site, comprising 14.4% of the total composition of the mosquito fauna at this site.

*Culex quinquefasciatus* breeds in fresh and polluted water and is often associated with drains, containers, and septic tanks (17). Calhoun et al. (48) showed that streams under the influence of nutrient pulses from sewage overflows had an increased presence of *C. quinquefasciatus*, and laboratory trials by Chaves et al. (49) found *C. quinquefasciatus* to oviposit in water with nutrients or sewage over tap water (where no oviposition occurred). However, the current study only found *C. quinquefasciatus* in 8.2% of backyard containers, perhaps influenced through the lack of nutrient load from reticulated water systems. Adult EVS CO_2_ traps collected *C. quinquefasciatus* in moderate numbers, perhaps indicating that drainage systems within residential areas are the preferred oviposition sites for female mosquitoes of this species compared to backyards. Water runoff may contain pollutants from vehicle traffic, herbicide, and fertilizer applications, perhaps creating a more suitable environment in underground drainage systems for this species. A comparison of water quality attributes between the storm water drainage infrastructure and of bore water most likely to have contributed to filling of backyard containers (supplied as a free resource to most Perth backyards) may provide a greater understanding of the development of mosquitoes associated with these features in urban environments.

Adult species composition differed markedly between the three habitats studied, which included residential backyards, Ashfield Flats, a tidal saltmarsh habitat and Bindaring Park, a small brackish creek with roadside runoff. Adult mosquitoes in domestic residential backyards were dominated by *A. notoscriptus*, as were the containers found associated with these residential lots. The saltmarsh mosquito species, *A. camptorhynchus* and *A. vigilax*, were only present in low densities within residential yards, indicating the greater public health risk associated with mosquito species breeding in backyard containers (principally *A. notoscriptus*). These findings were influenced by local environmental conditions prevalent at the time of the study, an El Niño weather pattern, reduced the number and magnitude of tides associated with the development of consecutive cohorts of saltmarsh species, often observed under La Niña weather patterns (50). In addition, chemical treatment of Ashfield Flats with s-methoprene briquettes may have substantially reduced adult saltmarsh mosquito populations developing within the tidally driven saltmarsh flats leading to a lower density of adult mosquitoes emerging from these sites and dispersing into the surrounding backyards of local residents.

This study was conducted within a single Local Government Authority within the eastern suburbs of Perth, WA, Australia. Further investigation of the role of backyard breeding should be conducted both within the Town of Bassendean as well as in other Local Government Authorities that border the Swan and Canning Rivers of Perth to determine if the trends observed are reflected across the Perth Metropolitan region. Further, repeated investigations of the same backyards may provide additional information in regards to the likelihood of residents recognizing backyard breeding and removing backyard containers to limit mosquito breeding. In addition, further investigation of backyard breeding should be completed over consecutive summers to determine if the low detection rate of backyard breeding observed in the current study was a result of the “dry” summer environmental conditions at the time of the study or rather a broad trend that could be observed across the region.

Local Government EHOs should aim to increase the awareness of backyard breeding and the need for property owners to reduce backyard containers capable of holding water in order to reduce the nuisance and disease risk associated with the common container-breeding mosquito species in the area. While both the State DoH and Local Government have deployed public education campaigns (largely as pamphlets and media statements) to residents in the area, the messages appear to be ineffective in ensuring residents maintain a yard free of container habitats that may be exploited by mosquitoes. The Town of Bassendean has since initiated a health promotion campaign with a series of stories being distributed to residents in local government publications and the creation of a Facebook page (Fatouros *personal communication* 2016). These initiatives could have a dramatic impact on the number of container habitats found around residential properties; particularly in El Niño years when natural breeding of saltmarsh mosquitoes are limited. Mosquito control efforts in “quiet mosquito seasons” should be directed at residential lots and the reduction of backyard container habitats, while in “busy seasons,” particularly under La Niña weather patterns, mosquito management needs to shift to the natural breeding sites of Ashfield Flats on the Swan River to reduce production of saltmarsh species.

While there has been a reduction in natural mosquito-breeding sites along the Swan and Canning Rivers, the increased density of residential housing and associated drainage infrastructure is leading to new challenges for mosquito management and the protection of the public from mosquito-borne diseases. As land-use planning continues to place privately owned residence within close proximity to low lying, tidal saltmarsh habitats along the Swan River, there will be a nuisance and possible disease risk associated with saltmarsh breeding mosquitoes. Under certain environmental conditions, predominantly La Niña weather systems the increased frequency and magnitude of tides, leads to repeated hatching of multiple cohorts of mosquitoes and this will remain the focus of Local Government control strategies. However, under El Niño weather patterns, the risks associated with nuisance and disease carrying mosquitoes appears to be mainly from domestic container-breeding species.

Although the current study only found 3% of containers to be actively breeding mosquitoes in residential backyards, 94% of residential backyards surveyed were found to possess at least one container habitat. These results indicate near universal capacity to sustain mosquito container breeding across residential backyards in the Town of Bassendean.

This study is only one step toward the development of an integrated mosquito management program for the Town of Bassendean in understanding the dynamics of mosquito breeding in complex urban areas and the establishment of an appropriate mosquito control program to protect public health.

## Ethics Statement

Prior to the collection of data, the study was carried out in accordance with the recommendations of the Edith Cowan University Human Research Ethics Committee (Ethics approval number 13843).

## Author Contributions

PN, JO, KC, and MF made substantial contribution to the design of the research. PN, RL, JO, KC, SM, and MF conducted field work for the collection and analysis of the data. PN, RL, JO, KC, MF, and SB contributed to the drafting of the manuscript and the important intellectual content herewith. RL, PN, JO, KC, SM, MF, and SB collaborated for the final version of the original research paper submitted and agreed to be accountable for all aspects of the work.

## Conflict of Interest Statement

The authors declare that the research was conducted in the absence of any commercial or financial relationships that could be construed as a potential conflict of interest.

## References

[1] Davis JA Froend R Loss and degradation of wetlands in southwestern Australia: underlying causes, consequences and solutions. Wetl Ecol Manag (1999) 7:13–23. 10.1023/A:1008400404021

[2] Morel-Ednie Brown F Layered landscape: the swamps of colonial Northbridge. Soc Sci Comp Rev (2009) 27(3):390–418. 10.1177/0894439308329765

[3] Hames S A Historical Geography of the Bayswater Foreshore, Swan River [Honours Thesis]. Edith Cowan University (2004). Available from: http://ro.ecu.edu.au/theses_hons/367

[4] Environmental Protection Authority. State of the Environment Report Western Australia Draft. Western Australia: Environmental Protection Authority (2006). Available from: http://www.bushlandperth.org.au/images/stories/PDF/Submissions/2006/State%20of%20the%20Environment%20Draft%20Report%20Sept%2006.pdf

[5] Grose M Hedgcock D Designs for stormwater disposal in public open space: an ecological assessment of current practices in Western Australia. Proceedings of the 1st National Hydropolis Conference Perth (2006). p. 123–42. Available from: https://www.researchgate.net/publication/240622562

[6] Staples K Oosthuizen J Lund M Effectiveness of S-Methoprene briquettes and application method for mosquito control in urban road gullies/catch basins/pots in a Mediterranean climate: implications for Ross River Virus transmission. J Am Mosq Con Ass (2016) 32(3):203–9. 10.2987/16-6563.1 27802404

[7] Staples K Evaluation of a Mosquito Control Intervention and Recommendations for Development of Best Practice Protocols by the Shire of Kalamunda [Masters Thesis]. Australia: Edith Cowan University (2016).

[8] McKiernan SP Docile Bodies of Water: Artificial Wetlands and Imagineered Suburbs [Honours Thesis]. Edith Cowan University (2001).

[9] Jennings ST Better Urban Water Management: A Case Study of Perth, WA [Thesis]. Murdoch University (2011).

[10] Blair A Control of Mosquitoes and Non-Biting Midges in Perth and Outer Urban Areas: A Report to the Western Australian Department of Conservation and Environment. Perth: Department of Conservation and Environment (1979). 75 p.

[11] Ballard JWO Marshall ID. An investigation of the potential of *Aedes camptorhynchus* (thom.) as a vector of Ross River Virus. Aust J Exp Biol Med Sci (1986) 64(2):197–200. 10.1038/icb.1986.21 3017279

[12] Lindsay MD Broom AK Wright AE Johansen CA Mackenzie JS. Ross River virus isolations from mosquitoes in arid regions of Western Australia: implication of vertical transmission as a means of persistence of the virus. Am J Trop Med Hyg (1993) 49(6):686–96. 10.4269/ajtmh.1993.49.686 8279636

[13] Lindsay MD Oliveira N Jasinska E Johansen C Harrington S Wright AE An outbreak of Ross River virus disease in Southwestern Australia. Emerg Infect Dis (1996) 2(2):117–20. 10.3201/eid0202.960206 8903211 PMC2639827

[14] Ryan AM Spash CL Measham TG Socio-economic and psychological predictors of domestic greywater and rainwater collection: evidence from Australia. J Hydro (2009) 379:164–71. 10.1016/j.jhydrol.2009.10.002

[15] Domènech L Sauri D A comparative appraisal of the use of rainwater harvesting in single and multi-family buildings of the Metropolitan Area of Barcelona (Spain): social experience, drinking water savings and economic costs. J Clean Prod (2011) 19:598–608. 10.1016/j.jclepro.2010.11.010

[16] Trewin BJ Kay BH Darbro JM Hurst TP. Increased container-breeding mosquito risk owing to drought-induced changes in water harvesting and storage in Brisbane, Australia. Int Health (2013) 5(4):251–8. 10.1093/inthealth/iht023 24225151

[17] Liehne PFS An Atlas of the Mosquitoes of Western Australia. Perth: Health Department of Western Australia (1991). 292 p.

[18] World Health Organization. Dengue: Guidelines for Diagnosis, Treatment, Prevention and Control. Geneva, Switzerland: WHO (2009). 160 p. 23762963

[19] Russell RC Bryan JH A survey of domestic container-breeding mosquitoes in New South Wales for the presence of *Aedes aegypti (L.)*, the vector of dengue fever. J Aust Entomol Soc (1985) 24:193–4. 10.1111/j.1440-6055.1985.tb00224.x

[20] Derraik JGB Mosquito breeding in container habitats in urban and peri-urban areas in the Auckland Region, New Zealand. Entomotropica (2005) 20(2):89–93. 10.1080/00779962.2005.9722691

[21] Chen CD Lee HL Stella-Wong SP Lau KW Sofian-Azirun M Container survey of mosquito breeding sites in a university campus in Kuala Lumpur, Malaysia. Dengue Bull (2009) 33:187–93.

[22] Rajesh K Dhanasekaran D Tyagi BK Survey of container breeding mosquito larvae (Dengue Vector) in Tiruchirappalli district, Tamil Nadu, India. J Entomol Zoo Stud (2013) 1(6):88–91.

[23] Adebote AD Oniye JS Ndams SI Nache KM The breeding of mosquitoes (Diptera: Culicidae) in peridomestic containers and implications for yellow fever transmission in villages around Zaria, Northern Nigeria. J Ento (2006) 3(2):180–8. 10.3923/je.2006.180.188

[24] Maciel-de-Freitas R Lourenço-de-Oliveira R. Does targeting key-containers effectively reduce *Aedes aegypti* population density? Trop Med Int Health (2011) 16:965–73. 10.1111/j.1365-3156.2011.02797.x 21605290

[25] Troyo A Calderón-Arguedas O Fuller DO Solano ME Avendaño A Arheart KL Seasonal profiles of *Aedes aegypti* (Diptera: Culicidae) larval habitats in an urban area of Costa Rica with a history of mosquito control. J Vector Ecol (2008) 33(1):76–88. 10.3376/1081-1710(2008)33[76:SPOAAD]2.0.CO;2 18697310 PMC2560178

[26] Bureau of Meteorology. Climate Statistics for Australian Locations – Perth Airport. (2017). Available from: www.bom.gov.au

[27] Sharma RS Kaul SM Sokhay J Seasonal fluctuations of dengue fever vector, *Aedes aegypti* (Diptera: Culicidae) in Delhi, India. Southeast Asian J Trop Med Pub Health (2005) 36:186–90. 15906665

[28] Vezzani D Albicócco AP. The effect of shade on the container index and pupal productivity of the mosquitoes *Aedes aegypti* and *Culex pipiens* breeding in artificial containers. Med Vet Entomol (2009) 23(1):78–84. 10.1111/j.1365-2915.2008.00783.x 19239617

[29] Potter A Jardine A Neville P. A survey of knowledge, attitudes, and practices in relation to Mosquitoes and Mosquito-Borne disease in Western Australia. Front Public Health (2016) 4:32. 10.3389/fpubh.2016.00032 26973827 PMC4770046

[30] Wong J Stoddard ST Astete H Morrison AC Scott TW. Oviposition site selection by the dengue vector *Aedes aegypti* and its implications for dengue control. PLoS Negl Trop Dis (2011) 5(4):e1015. 10.1371/journal.pntd.0001015 21532736 PMC3075222

[31] Bentley MD Day JF Chemical ecology and behavioural aspects of mosquito oviposition. Ann Rev Entomol (1989) 34:401–21. 10.1146/annurev.en.34.010189.002153 2564759

[32] Lee DJ Hicks MM Griffiths M Russell RC Marks EN The Culicidae of the Australasian Region, Vol 3. Entomology Monograph No 2. Canberra, Australia: Australian Government Publishing Service (1991).

[33] Blaustein L Kotler BP Oviposition habitat selection by the mosquito, *Culiseta longiareolata*: effects of conspecifics, food and green toad tadpoles. Ecol Entomol (1993) 18(2):104–8. 10.1111/j.1365-2311.1993.tb01190.x

[34] Kiflawi M Blaustein L Mangel M Oviposition habitat selection byu the mosquito *Culiseta longiareolata* in response to risk of predation and conspecific larval density. Ecol Entomol (2003) 28:168–73. 10.1046/j.1365-2311.2003.00505.x

[35] Blaustein L Blaustein J Chase J. Chemical detection of the predator *Notonecta irrorata* by ovipositing *Culex* mosquitoes. J Vector Ecol (2005) 30(2):299–301. 16599167

[36] Dhileepan K Physical factors and chemical cues in the oviposition behaviour of arboviral vectors *Culex annulirostris* and *Culex molestus* (Diptera: Culicidae). Environ Entomol (1997) 26(2):318–26. 10.1093/ee/26.2.318

[37] Mokany A Shine R Oviposition site selection by mosquitoes is affected by cues from conspecific larvae and anuran tadpoles. Austral Ecol (2003) 28:33–7. 10.1046/j.1442-9993.2003.01239.x

[38] Watson TM Kay BH. Vector competence of *Aedes notoscriptus* (Diptera: Culicidae) for Ross River virus in Queensland, Australia. J Med Entomol (1998) 35(2):104–6. 10.1093/jmedent/35.2.104 9538569

[39] Verdonschot P Besse-Lotoskaya A Flight distance of mosquitoes (Culicidae): a metadata analysis to support the management of barrier zones around rewetted and newly constructed wetland. Limnilogica Ecol Manag Inland Waters (2014) 45:69–79. 10.1016/j.limno.2013.11.002

[40] Claflin SB Webb CE Ross river virus: many vectors and unusual hosts make for an unpredictable pathogen. PLoS Pathog (2015) 11(9):e1005070 10.1371/journal.ppat.1005070 26335937 PMC4559463

[41] Derraik JGB Slaney D Influence of container aperture size and colour on oviposition preferences in three New Zealand mosquitoes (Diptera Culicidae). Ann Med Entomol (2005) 14:30–41.

[42] Derraik JGB Slaney D. Container aperture size and nutrient preferences of mosquitoes (Diptera: Culicidae) in the Auckland region, New Zealand. J Vector Ecol (2005) 30(1):73–82. 16007958

[43] Warchot A Whelan P Biting Insect Report for the Darwin City Waterfront Redevelopment. Northern Territory: Department of Health and Community Services (2004).

[44] Mackenzie JS Lindsay MD Coelen RJ Broom AK Hall RA Smith DW. Arboviruses causing human disease in the Australasian zoogeographic region. Arch Virol (1994) 136:447–67. 10.1007/BF01321074 8031248

[45] Russell RC Ross River virus: disease trends and vector ecology in Australia. Bull Soc Vector Ecol (1994) 19:73–81.

[46] Russell RC Arboviruses and their vectors in Australia: an update on the ecology and epidemiology of some mosquito-borne arboviruses. Rev Med Vet Entomol (1995) 83:141–58.

[47] Johansen CA Power SL Broom AK Determination of mosquito (Diptera: Culicidae) bloodmeal source in Western Australia: implications for arbovirus transmission. J Med Entomol (2009) 46(5):1167–75. 10.1603/033.046.0527 19769051

[48] Calhoun LM Avery M Jones L Gunarto K King R Roberts J Combined sewage overflows (CSO) are major urban breeding sites for *Culex quinquefasciatus* in Atlanta, Georgia. Am J Trop Med Hyg (2007) 77(3):478–84. 17827363

[49] Chaves LF Keogh CL Vazquez-Prokopec GM Kitron U. Combined sewage overflow enhances oviposition of *Culex quinquefasciatus* (Diptera: Culicidae) in Urban Areas. J Med Entomol (2009) 46(2):220–6. 10.1603/033.046.0206 19351072

[50] Kelly-Hope LA Purdie DM Kay BH Ross River virus disease in Australia, 1886–1998, with analysis of risk factors associated with outbreaks. J Med Entomol (2004) 41(2):133–50. 10.1603/0022-2585-41.2.133 15061271

